# Abnormal white matter structural connectivity in adults with obsessive-compulsive disorder

**DOI:** 10.1038/tp.2017.22

**Published:** 2017-03-14

**Authors:** J Gan, M Zhong, J Fan, W Liu, C Niu, S Cai, L Zou, Ya Wang, Yi Wang, C Tan, R C K Chan, X Zhu

**Affiliations:** 1 Medical Psychological Center, the Second Xiangya Hospital, Central South University, Changsha, Hunan, China; 2 Medical Psychological Institute of Central South University, Changsha, Hunan, China; 3 Center for Studies of Psychological Application, School of Psychology, South China Normal University, Guangzhou, China; 4 Department of Radiology, The Second Xiangya Hospital, Central South University, Changsha, China; 5 Neuropsychology and Applied Cognitive Neuroscience Laboratory, CAS Key Laboratory of Mental Health, Institute of Psychology, Chinese Academy of Sciences, Beijing, China; 6 University of Chinese Academy of Sciences, Beijing, China

## Abstract

Obsessive-compulsive disorder (OCD) is a complex and severe psychiatric disorder whose pathogenesis is not fully understood. Recent studies have shown white matter (WM) alterations in adults with OCD, but the results have been inconsistent. The present study investigated WM structure in OCD patients with the hypothesis that large-scale brain networks may be disrupted in OCD. A total of 24 patients with OCD and 23 healthy controls (HCs) were scanned with diffusion tensor imaging. A tract-based spatial statistics (TBSS) approach was used to detect differences across the whole brain in patients with OCD vs HCs; *post hoc* fiber tractography was applied to characterize developmental differences between the two groups. Relative to HCs, patients with OCD had lower fractional anisotropy (FA) values in the corpus callosum (CC), left anterior corona radiata (ACR), left superior corona radiata (SCR) and left superior longitudinal fasciculus (SLF), and higher radial diffusivity in the genu and body of CC. Among the TBSS de-projected region of interest results, compared with HCs, patients with OCD showed lower of the mean FA values of fiber bundles passing though the SLF, and shorter lengths of ACR, SCR and CC. In conclusion, this study provides novel evidence of widespread microstructural alterations in OCD and suggests that OCD may involve abnormalities affecting a broader network of regions than commonly believed.

## Introduction

Obsessive-compulsive disorder (OCD) is a common mental disorder affecting 2~3% of the general population internationally.^1^ Recurrent, intrusive and distressing thoughts and/or repetitive behaviors are regarded as the core difficulties for patients with OCD;^2^ impaired attention and spatial working memory abnormalities are also common in OCD patients.^3, 4^ These clinical features predispose patients to impairments of occupational and social functioning.

Neurobiologically, the aforementioned core elements of OCD have been linked to dysfunction of cortico-striatal circuits.^5^ The cortico-striatal circuitry model of OCD suggests that the hallmark of OCD is a dysfunction of a neuronal loop running from the orbitofrontal and cingulate cortices to the striatum (caudate nucleus and putamen), globus pallidus, thalamus and back to the frontal cortex.^6^ Among frontal regions, the dorsolateral prefrontal cortex, oritofrontal cortex and anterior cingulate cortex seem to be of major psychopathological relevance to OCD.^5, 7, 8^ This conceptualization of cortico-striatal dysfunction in OCD has spurred structural and functional imaging research, focusing mainly on the orbitofrontal cortex, cingulate cortex, striatum and thalamus, and temporolimbic regions (that is, lateral and medial temporal cortices, amygdala, hippocampus, parahippocampal cortex, hypothalamus and insular cortex).^9^ More recently, whole-brain analyses also have implicated additional parietal, insular and cerebellar regions.^9, 10^ These findings may reflect abnormalities in white matter (WM) tracts, which can affect connectivity between diverse brain regions.^11^

An increasing number of studies using diffusion tensor imaging (DTI) are suggesting that the OCD brain has WM alterations apart from previously described alterations in gray matter regions.^12^ DTI is a noninvasive method that can be used to probe the intrinsic, three-dimensional diffusion properties of water within tissues; it is sensitive to the orientation and integrity of underlying WM fibers *in vivo*.^13^ In the DTI paradigm, fractional anisotropy (FA) is an index of diffusion directionality in each voxel,^14^ which can reflect the structure of axonal cell membranes and myelin sheaths, with high FA values being observed in heavily myelinated tracts.^15^ Mean diffusivity (MD) is the magnitude of diffusion in each measured voxel, which varies with tissue density regardless of fiber orientation.^16^ Axial diffusivity (AD) is the tendency to diffuse along the principal direction of the fiber, and radial diffusivity (RD) is the diffusivity perpendicular to axonal walls. Changes in RD appears to be associated with dys- or demyelination (cell membrane and cytoskeleton) in WM, whereas variations in AD may be more specific to axonal degeneration (volume and organization).^17^

DTI based on voxel based morphometry (VBM) can be used to investigate WM abnormalities within as well as beyond the cortico-striatal circuits classically associated with OCD.^18^ Studies of OCD using whole-brain and selected region of interest (ROI) analyses have revealed variable FA values in the anterior cingulate, internal capsule, bilateral semioval center extending to medial frontal WM, subinsular WM and corpus callosum (CC), as well as in WM in parietal,^18, 19, 20^ left lingual gyrus and occipital lobe^20^ regions. However, these prior results are rather heterogeneous. For example, FA values in OCD brains have been reported to be both increased and decreased, in some cases in the same brain areas.^4, 11, 18, 19, 20, 21, 22, 23, 24, 25, 26^ The heterogeneous findings might be due to limitations of voxel-based morphometry methods, which do not yield accurate inter-subject image registration.^14^

Tract-based spatial statistics (TBSS) is an analytic method developed specifically for DTI data wherein analysis is restricted to center areas of major white-brain volumes.^27^ This feature makes TBSS highly sensitive to changes in microstructure within the major WM fiber pathways. The only three studies to have used TBSS to analyze DTI in OCD adults^11, 22, 28^ all found significantly reduced FA values, relative to controls, in the body of the CC. However, comprehensive MD, AD, and RD data reporting from these studies was lacking.

Importantly, TBSS analysis minimizes the potential misalignment problems of other voxel-based whole-brain analysis methods by producing a WM ‘skeleton' restricted to the center of major WM tracts, and mapping diffusion values from each individual participant directly onto this standard skeleton for group comparisons. However, TBSS analysis fails to reveal commissural connections to lateral cerebral areas, probably because of massive projection and long association fibers being situated lateral to the CC, beyond the reach of reconstruction tracking. Previous DTI studies have focused exclusively on hypothesis-driven ROIs or broad exploratory TBSS analysis; both approaches are important and the two are complementary. Only two studies conducted *post hoc* tractography analysis based on TBSS results by probabilistic tracking; they showed CC connections to the bilateral dorsal medial frontal cortex,^14, 22^ dorsolateral prefrontal cortex^22^ and parietal regions as well as connections extending down to superior temporal regions,^14^ but neither reported further analyses on these commissural fiber tracts, such as the FA values or lengths.

In the present study, we examined WM microstructure in patients with OCD compared with age-, handedness- and education-matched healthy control (HC) participants. Utilizing TBSS, we examined four diffusivity measures (FA, MD, AD and RD) within major WM pathways to explore whether abnormalities of large-scale brain systems were detectable in the brains of patients with OCD. Using *post hoc* ROI tractography, we also examined anatomical connectivity via association and commissural fibers, which have not been investigated specifically previously.

## Materials and methods

### Participants and psychiatric measures

A group of 24 OCD outpatients (Table 1) was recruited from an outpatient clinic at the Second Xiangya Hospital of Central South University in Changsha, Hunan, China, from September 2014 to August 2015. All 24 patients were diagnosed with OCD and screened for other major psychiatry disorders by two experienced psychiatrists based on the Chinese version of the Structured Clinical Interview for the Diagnostic and Statistical Manual of Mental Disorders, 4th edition Axis I. Patients were excluded for major psychiatric and behavioral disorders such as schizophrenia, schizoaffective disorder, major depression disorder, bipolar disorder and autism spectrum disorder as well as for alcohol/drug dependence, eating disorders, significant neurological diseases or a family history of major psychiatric disorders. The presence of physical disorders with known psychiatric consequences (for example, hypothyroidism, seizure disorder or brain injury) was also an exclusion criterion. Severity of OCD symptoms (OCD group) was assessed with the Yale-Brown obsessive-compulsive scale (revised),^29^ and general intelligence of all the participants was assessed with the Wechlser Abbreviated Scale of Intelligence.^30^ Final diagnoses were determined by the consensus of psychiatrists. In the 4 weeks preceding DTI, 11 OCD patients had been free of any psychotropic medication and 13 were taking antidepressants (sertraline hydrochloride, *N*=10; venlafaxine, *N*=1; mirtazapine, *N*=1; and paroxetine, *N*=1).

A group of 23 age-, sex-, intelligence quotient- and handedness-matched HCs (Table 1) from the local community were recruited and screened for psychiatric and behavioral disorders by two experienced psychiatrists using the Structured Clinical Interview for Diagnostic and Statistical Manual of Mental Disorders, 4th edition Axis I. None were found to have any neurological and psychiatric illnesses, major physical diseases or positive family history of major psychiatric disorders. Their general intelligence was also assessed with the Wechlser Abbreviated Scale of Intelligence.^30^

The study protocol was designed in accordance with the guidelines outlined in the Declaration of Helsinki and was approved by the Ethics Committee of the Second Xiangya Hospital of Central South University. All the subjects were made aware of the purpose of the study and provided written informed consent.

### Magnetic resonance imaging

All the participants were subjected to magnetic resonance imaging in a Siemens Magnetom Skyra 3.0-T scanner (Erlangen, Germany). A T1-weighted sagittal isotropic magnetization-prepared rapid acquisition gradient-echo sequence was obtained for each subject with a standard head coil (repetition time=1900 ms, echo time=2.01 ms, field of view=256 mm, slice thickness=1.0 mm and voxel size=1.0 × 1.0 × 1.0 mm^3^). A single-shot echo-planar imaging sequence was applied for DTI assessment (repetition time=6400 ms, echo time=86 ms, field of view 256 mm, 96 × 93 matrix size). Each scan produced 55 slices (thickness=2.5 mm, no gap) and 76 contiguous axial slices (64 gradient directions, two *b* values (0 and 1000 s mm^−2^)). Both the sequences were reviewed by an experienced radiologist to exclude clinical abnormalities. All the participants were asked to remain quiet during scanning. Ear plugs and foam pads were used to minimize noise exposure and head movements.

### TBSS analysis

Analyses of diffusion-weighted images were done in FMRIB'S Software Library (FSL, http://www.fmrib.ox.ac.uk/fsl). Eddy Current Correction was performed by affine registration to a b0 reference volume. A diffusion tensor model was fitted to each voxel, and then used to generate FA, MD, AD and RD values. The diffusion-weighted images were registration to the BET applied structural (T1-weighted) images. TBSS was used to carry out voxel-wise analysis for relevant diffusivity measures in WM skeleton voxels (FA >0.2).

The differences in FA, MD, AD and RD values between the OCD and HC groups, and between medicated and treatment-naive OCD subgroups, were detected by voxel-wise independent two-sample *t*-tests in Randomize (*n*=10 000 repetitions; confidence threshold of *P*<0.05 for FWE-corrected). Finally, the FA values were extracted from the significant clusters via the ‘fslmeants' command.

### Fiber tractography

Reconstruction and fiber tracking analysis of the DTI data were performed in Diffusion Toolkit software (http://www.trackvis.org/), and ROI drawing and visualization of the results were performed in TrackVIS software (http://www.trackvis.org/). The FACT approach was used to reconstruct fiber paths. Fiber propagation was terminated when fiber orientation changed by an angle ⩾35°, and an image mask based on the B0 image was applied to restrict tracking to biologically plausible results.

**Figure 1:**
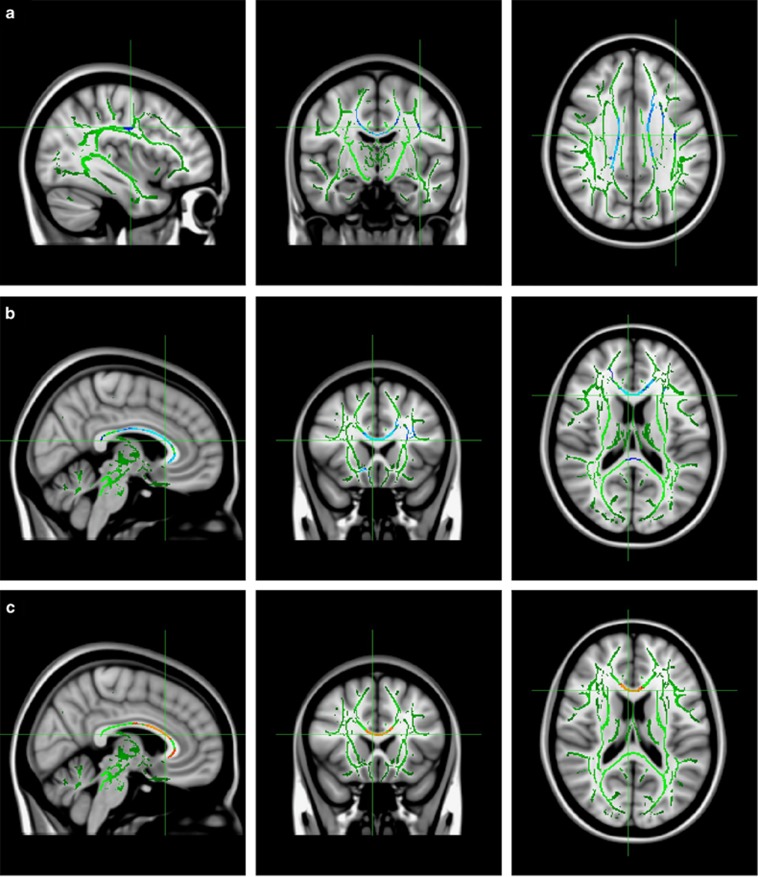
Group differences in FA and RD. WM structures showed decreased FA (blue) in (**a**) the left superior longitudina fasciculus (SLF; MNI *x*, *y*, *z*: 127, 115, 104), (**b**) the splenium, body and genu of CC, including the left anterior and superior corona radiate (ACR and SCR; MNI *x*, *y*, *z*: 85, 146, 88) and increased RD (red) in (**c**) the body and genu of the CC (MNI *x*, *y*, *z*: 85, 146, 89) in patients with OCD (*P*<0.05 vs HCs, corrected for multiple comparisons). FA maps show sagittal, coronal and axial views (from left to right). The background image is a standard MNI-1521-mm brain template. Green voxels represent the FA WM skeleton. Red–yellow voxels represent regions with significantly lower FA, and light blue–blue voxels represent regions with significantly higher RD in the OCD group vs HCs. The TBSS_till script was implemented in FMRIB's Software Library. CC, corpus callosum; FA, fractional anisotropy; HC, healthy control; MNI, Montreal Neurological Institute atlas; OCD, obsessive-compulsive disorder; RD, radial diffusivity; TBSS, tract-based spatial statistics; WM, white matter.

To define fiber tractography seed points as the ROIs, clusters identified as differing (*P*<0.05, FWE-corrected) between the two groups in the TBSS analysis were de-projected in native space by the back-projection procedure (tbss_deproject script) in TBSS (Figure 1a). Individual ROIs were loaded into TrackVIS software to calculate the FA values of tracts passing through them as well as the numbers and average lengths of fibers within those passages (Figure 1b).

### Statistical analysis

Group differences on demographic and psychometric characteristics were compared with the chi-square test for categorical variables and independent-sample *t*-tests for continuous variables. Group comparisons of FA values, fiber numbers and fiber bundle lengths of the tracts passing through the ROIs derived from the TBSS results were completed with independent-sample *t*-tests. All statistical analyses were carried out in SPSS 18.0 (SPSS, Chicago, IL, USA).

## Results

### Demographic and psychometric data

The characteristics of the OCD and HC groups are summarized in Table 1. There were no significant differences between the two groups in terms of age or intelligence quotient scores (Table 1).

### Imaging findings

TBSS analysis revealed that the OCD group exhibited three clusters with significantly lower FA values than those of the HC group, mainly in the regions of the left superior longitudinal fasciculus (SLF), left anterior corona radiata (ACR), left superior corona radiata (SCR) and CC (including the splenium, genu and body; Table 2, Figure 1, Supplementary Table 1). Meanwhile, relative to HCs, the OCD group had higher RD, particularly in the genu and body of CC (Table 2, Figure 1, Supplementary Table 1). No regions had significantly higher FA or lower RD in the OCD group relative to the HC group. There were no significant group differences in the MD or AD values in any regions. There were also no significant FA differences in any regions between the medicated group and treatment-naive subgroups of patients with OCD.

**Figure 2:**
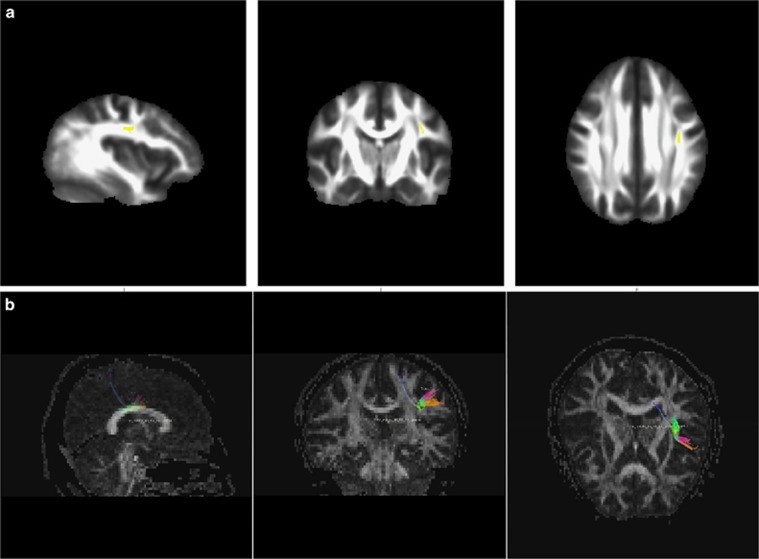
Fiber tracking. (**a**) ROIs from TBSSs were back-projected to the native space in sagittal, coronal and axial planes. (**b**) Fiber tracking through ROIs of superior longitudina fasciculus group comparison of FA values of tracts passing through the ROI of superior longitudina fasciculus (*P<0.05). FA, fractional anisotropy; ROI, region of interest; TBSS, tract-based spatial statistics.

**Figure 3:**
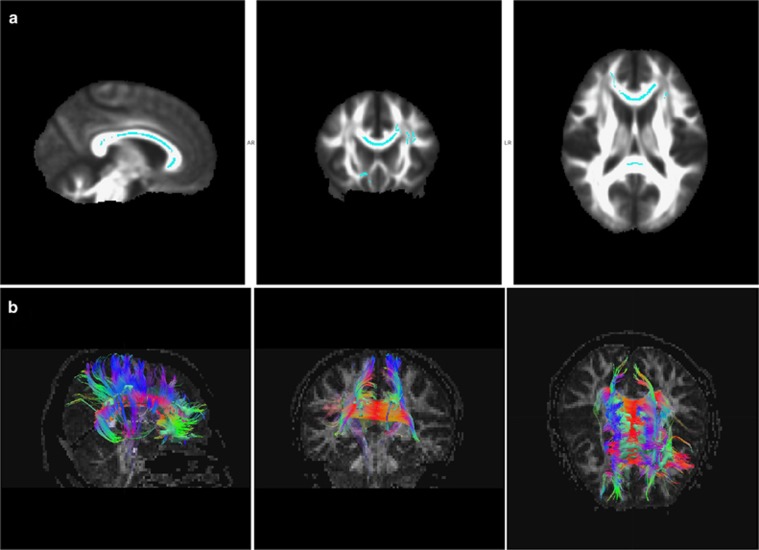
Fiber tracking. (**a**) Regions of interest (ROIs) from tract-based spatial statistics (TBSS) results were back-projected to the native space in sagittal, coronal and axial planes. (**b**) Fiber tracking passing through ROIs of the splenium, body and genu of corpus callosum (CC; including the left anterior and superior corona radiate).

Among the TBSS de-projected ROI results (Figures 2 and 3), only the mean FA values of fiber bundles passing through the SLF were significantly lower in the OCD group (FA=0.52±0.03) than in the HC group (FA=0.55±0.03; *P*=0.012), but no significant group differences were found for the length of these fiber bundles (length_OCD_=42.69±11.72; length_HC_=49.60±13.40; *P*=0.067). Notably, FA values of fiber bundles passing through the ACR, SCR and CC (splenium, genu and body) did not differ between the two groups (FA_OCD_=0.59±0.02; FA_HC_=0.60±0.02; *P*=0.078), but the lengths of these fiber bundles were significantly shorter in the OCD group (length_OCD_=48.67±5.15) than in the HC group (length_HC_=52.25±6.51; *P*=0.043). No significant group differences were found for tract numbers, voxel numbers or volumes of fiber bundles passing through the ROIs (Supplementary Table 1).

Power analysis predicted a detectable effect size (Cohen's *d*) at 0.737 when comparing differences between HCs (*N*=23) and OCD patients (*N*=24) with 80% power at a 5% significance level (with G-power, version 3.1.9.2). The actual Cohen's *d* values obtained for between-group differences ranged from 0.853 to 1.169, all of which exceeded the predicted detectable effect size, indicating that the sample size was acceptable.

## Discussion

In this study, exploratory TBSS analysis aimed at investigating potential subtle WM changes in association with OCD revealed OCD-associated deficits in important WM tracts, including within the CC and SLF. To the best of our knowledge, this study was the first to use TBSS and *post hoc* tractography to obtain an integrative view of WM alterations in OCD, including potential alterations affecting connections of differing major fibers and lateral cerebral areas which had not been considered in previous DTI-based studies of OCD.

Consistent with prior evidence,^31, 32^ the location of abnormal WM in the splenium, genu and body of the CC, ACR and SCR implicates abnormal function and structure of frontal–striatal–thalamic circuitry. The genu connects the lateral and medial surfaces of the frontal lobes^33^—areas described as having abnormal functioning and volumes in previous studies of OCD. The splenium of the CC connects the occipital and inferior temporal regions,^10, 33^ where abnormally increased resting state metabolism and hyperactive responses to cognitive demands have been observed.^34^ The CC body provides broad connections among neocortical homotopic regions, including the premotor, supplementary motor, motor, somatesthetic and posterior parietal regions.^22, 33^ The ACR and SCR encompass projection fibers that converge in the internal capsule, either between the thalamus and putamen or between the caudate and putamen, and then approach the cortex.^35^ Thus, collectively, low FA values for the splenium, CC genu and body, ARC and SCR are suggestive of compromised integrity in key major WM tracts connecting frontal–striatal–thalamic circuitry nodes in neurocircuitry models of OCD. Radua *et al.*^36^ suggested that decreased FA in WM structures may suggest increased fiber crossing or, alternatively, may indicate disrupted or reduced myelination in those regions or perhaps decreased fiber density or coherence.

Combining TBSS and fiber tractography methods, we found FA alterations and changes of lengths in the CC, ACR and SCR of OCD brains, relative to HC brains, without volume or voxels differences. Previously, FA reductions have been attributed to changes in membrane permeability or the presence of non-axonal components, such as other cells, vessels or interstitial fluid.^36^ Consistent with the findings of Bora *et al.,*^11^ our current analysis revealed that RD was increased while AD was unchanged in the CC of patients with OCD, compared with HCs. Maps of parallel diffusivity (thought to represent myelination or axon packing density)^37^ support such speculation. Hence, our findings suggest possible demyelination in the commissural fibers of patients with OCD. Previous TBSS studies, whole brain or of the CC as an ROI, have often not revealed commissural connections to lateral cerebral areas.^11, 22, 28^ In the current study, we found shorter corticopetal CC fiber lengths in the OCD group, compared with those in HCs, but commissural fiber tract FA values did not differ significantly between the two groups. These shorter than typical fiber bundles might constrain or augment local information processing within these regions, with reduced transmission to other larger areas. In this context, compromised WM integrity and abnormally short fiber tracts may result in hypo-transmission of information, particularly from the neocortex to the paleocortex. This altered connectivity could facilitate neuronal integration between limbic and motor systems, while also providing additional opportunities for fine-tuning of striatal processing through feedback from areas in the cortex and mesencephalon.^38^ Such changes might underlie, at least in part, the cognitive dysfunction and abnormal behavior characteristic of OCD. If so, the brain abnormalities underlying OCD symptoms may be reflected throughout a broad network (that is, frontal–striatal–thalamic circuitry).

The present results also showed reduced FA values in the SLF of OCD brains, relative to HC brains. The SLF encompasses a heterogeneous set of bi-directional fibers connecting parieto-temporal association areas with prefrontal cortex areas.^39^ Impaired attention and spatial working memory, which have been documented in OCD patients,^3, 4^ may be consequent to disturbances in SLF connectivity. Gariboto *et al.*^4^ found evidence of involvement of occipital and parietal regions in the clinical phenomenology of OCD, including distressful, intrusive imagery. In their recent review, Piras *et al.*^10^ highlighted that OCD-related brain abnormalities are not limited to the affective orbitofronto–striatal circuitry, but rather extend to dorsolateral prefronto–striatal executive cirtcuitry as well as to regions in the parietal, temporal and occipital lobes.^10, 36^ Our findings confirm the involvement of executive circuit regions in OCD and further suggest the presence of multiple WM tract abnormalities in the brains of adults with OCD. However, as voxel size of left SLF was relatively small, the significance of their representations still needs to be further confirmed, even the findings in SLF were statistically significant.

Previous TBSS-based studies of OCD have yielded evidence of abnormalities in the CC,^3, 14, 22, 28^ inferior and superior longitudinal fasciculus,^3, 28, 40^ cingulum bundle^22, 41^ and internal capsule.^3, 22^ Meanwhile voxel-based morphometry/ROI-based studies of OCD have reported mostly changes in the striatum; thalamus; and frontal, parietal, temporal and cingulate cortices.^10^ In this study, we did not observe any evidence of WM deficits in the frontal, parietal, temporal or occipital lobes. These inconsistencies between studies might be due to the fact that TBSS analysis assesses only the most common fiber pathways across individuals, typically excluding fibers penetrating the cortical mantle. Thus, with the TBSS approach, sensitivity in regions near the gray matter/WM boundaries may be limited. Alternatively, structurally compromised WM may be reflected in low FA values, while hyper-connected regions—perhaps produced in compensation for network hypo-connectivity—may yield increased FA values in some ROI studies. If so, such compensatory changes may become undetectable with the strict FWE correction in TBSS analyses, leaving only significant deficits.

This study had several limitations. First, we did not collect behavioral data, preventing us from relating WM changes directly to clinical behavior. Task-related neuroimaging studies are needed to examine how WM connectivity relates to cognitive function in OCD patients. Second, our OCD group included both medicated and treatment-naive participants. There were no significant differences observed between these two subgroups. However, given the relatively small size of the sample and the variety of medication types and doses among the medicated patients, it remains unclear whether DTI results in OCD patients are affected by pharmacological treatment. Finally, we did not account for age of OCD onset in our recruitment or analysis. It is possible that WM alterations may evolve over time in relation to OCD onset.

In conclusion, the present study showed widespread microstructural alterations in the brains of patients diagnosed with OCD, predominantly in the CC and SLF, areas which may have important roles in OCD pathogenesis. Our findings suggest that OCD may involve abnormalities affecting a broader network of regions than commonly believed, that is, beyond the frontal–striatal–thalamic circuitry. Longitudinal studies are needed to elucidate the underlying mechanisms of WM changes in patients with OCD.

## Figures and Tables

**Table 1:** Demographic and clinical characteristics of the study participants

*Characteristic*	*Group, mean±s.d. or* n*%*		*Group comparisons*	
	*OCD (*N*=24)*	*HC (*N*=23)*	t*/*χ^*2*^	P
Age	22.58±6.56	23.17±2.12	−0.419	0.679
Females:males	9 (37.5%):15 (63.5%)	11 (91.7%):12 (8.3%)	0.512	0.474
IQ	129.64±16.32	137.27±20.60	1.405	0.168
BDI	21.00±16.20	5.57±5.82	−4.308	<0.001
STAI-S	53.71±11.48	36.23±10.71	−5.326	<0.001
STAI-T	56.46±9.87	39.91±10.69	−5.516	<0.001
YBOCS	32.45±5.98	—	—	—

Abbreviations: BDI, Beck Depression Inventory; HC, healthy control; IQ, intelligence quotient; OCD, obsessive-compulsive disorder; STAI-S, State Subscale of the State-Trait Anxiety Inventory;

STAI-T, Trait Subscale of the State-Trait Anxiety Inventory; YBOCS, Yale-Brown Obssesive Compulsive Scale.

**Table 2:** MNI coordinates of regions with decreased FA and increased RD in OCD group relative to HCs

*Variable*	*TFCE FWE-correlate* *P*	*MNI coordinates*			*Voxel size*	*Tract(s) within clusters*
		x	y	z		
*FA*						
Cluster 1	<0.05	127	115	104	76	Left of superior longitudina fasciculus
Cluster 2	<0.005	85	146	88	6357	Splenium, body and genu of CC (including left of anterior and superior corona radiata)
						
*RD*						
Cluster 1	<0.02	85	146	89	1543	Body and genu of CC

Abbreviations: CC, corpus callosum; FA, fractional anisotropy; FWE, family-wise error rate; HC, healthy control; MNI, Montreal Neurological Institute atlas; OCD, obsessive-compulsive disorder; RD, radial diffusivity; TFCE, threshold-free cluster enhancement.

*P*=0.05.
