# Supplementary material for: Abnormal white matter structural connectivity in adults with obsessive-compulsive disorder
Source: Transl Psychiatry. 2017 Mar 14;7(3):e1062–. doi: 10.1038/tp.2017.22 (PMC5416668; doi:10.1038/tp.2017.22)
Supplement: Supplementary Table S1 [file tp201722x1.docx]

Table S1 Supplementary of the imaging findings.

| Cluster | OCD group | HC group | t value | P |
| --- | --- | --- | --- | --- |
| SLF_FA_TBSS | 0.5303 ± 0.0319 | 0.5707 ± 0.0430 | 3.645 | .001 |
| CC_FA_TBSS | 0.6122 ± 0.0244 | 0.6422 ± 0.0155 | 5.017 | .000 |
| CC_RD_TBSS | 0.0004 ± 0.00004 | 0.0003 ± 0.00002 | -5.210 | .000 |
| SLF_track_dtk | 111.29 ± 35.71 | 126.22 ± 37.79 | - | - |
| ROIs1_voxel_dtk | 370.88 ± 139.73 | 552.09 ± 553.45 | - | - |
| ROIs1_volume_dtk | 3.71 ± 1.40 | 5.94 ± 7.70 | - | - |
| ROIs1_length_dtk | 42.69 ± 11.72 | 49.60 ± 13.40 | - | - |
| ROIs1_FA_dtk | 0.5246 ± 0.0308 | 0.5474 ± 0.0290 | 2.617 | .012 |
| ROIs2_track_dtk | 4272.09 ± 456.34 | 4578.09 ± 654.33 | - | - |
| ROIs2_voxel_dtk | 9591.92 ± 1020.83 | 10087.00 ± 1444.68 | - | - |
| ROIs2_volume_dtk | 95.91 ± 10.21 | 100.87 ± 14.445 | - | - |
| ROIs2_length_dtk | 48.67 ± 5.15 | 52.25 ± 6.51 | 2.088 | .043 |
| ROIs2_FA_dtk | 0.6471 ± 0.0326 | 0.6935 ± 0.0390 | - | - |

**_TBSS: Clusters identified as differing (*p* < .05, FWE-corrected) between the two groups in TBSS analysis;

**_dtk: Fiber tracts passing through ROIs in fiber tractography analysis
